# Di-μ-cyanido-tetra­cyanido(5,5,7,12,12,14-hexa­methyl-1,4,8,11-tetra­aza­cyclo­tetra­decane)[*N*-(quinolin-8-yl)quinoline-2-carboxamidato]diiron(III)nickel(II) 2.07-hydrate

**DOI:** 10.1107/S1600536813010234

**Published:** 2013-04-20

**Authors:** Yuqi Yang, Hongbo Zhou, Xiaoping Shen

**Affiliations:** a School of Chemistry and Chemical Engineering, Jiangsu University, Zhenjiang 212013, People’s Republic of China

## Abstract

The asymmetric unit of the title complex, [Fe_2_Ni(C_19_H_12_N_3_O)_2_(CN)_6_(C_16_H_36_N_4_)]·2.07H_2_O, contains one [Fe(qcq)(CN)_3_]^−^ anion, half a [Ni(teta)]^2+^ cation and two partially occupied inter­stitial water mol­ecules [qcq^−^ is the *N*-(quinolin-8-yl)quinoline-2-carboxamidate anion and teta is 5,5,7,12,12,14-hexa­methyl-1,4,8,11-tetra­aza­cyclo­tetra­deca­ne]. In the complex mol­ecule, two [Fe(qcq)(CN)_3_]^−^ anions additionally coordinate the central [Ni(teta)]^2+^ cation through cyanide groups in a *trans* mode, resulting in a trinuclear structure with the Ni^2+^ cation lying on an inversion centre. The two inter­stitial water mol­ecules are partially occupied, with occupancy factors of 0.528 (10) and 0.506 (9). O—H⋯O and O—H⋯N hydrogen bonding involving the two lattice water molecules and the carbonyl function and a teta N atom in an adjacent cluster leads to the formation of layers extending parallel to (010).

## Related literature

For the synthesis and background to low-dimensional systems based on modified hexa­cyanido­metalates, see: Liu *et al.* (2010 ▶); Kim *et al.* (2009 ▶); Curtis *et al.* (1964 ▶). For related structures, see: Li *et al.* (2012 ▶); Panja *et al.* (2012 ▶).
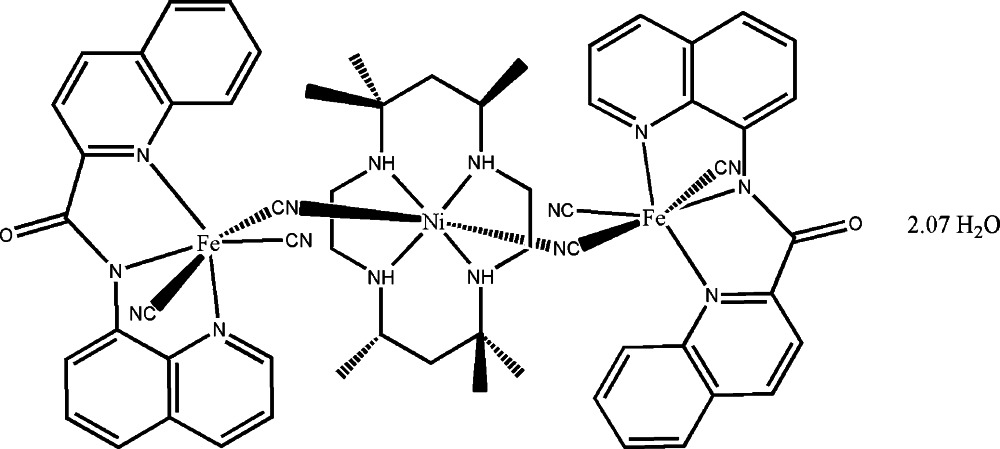


## Experimental

### 

#### Crystal data

[Fe_2_Ni(C_19_H_12_N_3_O)_2_(CN)_6_(C_16_H_36_N_4_)]·2.07H_2_O
*M* *_r_* = 1244.89
Monoclinic, 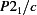

*a* = 9.4145 (13) Å
*b* = 15.7309 (17) Å
*c* = 20.590 (2) Å
β = 101.781 (3)°
*V* = 2985.1 (6) Å^3^
*Z* = 2
Mo *K*α radiation
μ = 0.85 mm^−1^
*T* = 291 K
0.28 × 0.24 × 0.22 mm

#### Data collection

Rigaku Saturn 724 CCD diffractometer
Absorption correction: multi-scan (*ABSCOR*; Higashi, 1995 ▶) *T*_min_ = 0.796, *T*_max_ = 0.835
12764 measured reflections
5722 independent reflections
4078 reflections with *I* > 2σ(*I*)
*R*_int_ = 0.022

#### Refinement

*R*[*F* ^2^ > 2σ(*F* ^2^)] = 0.057
*wR*(*F* ^2^) = 0.156
*S* = 0.97
5722 reflections
402 parameters
7 restraints
H atoms treated by a mixture of independent and constrained refinement
Δρ_max_ = 0.42 e Å^−3^
Δρ_min_ = −0.42 e Å^−3^

### 

Data collection: *CrystalClear* (Rigaku, 2008 ▶); cell refinement: *CrystalClear*; data reduction: *CrystalClear*; program(s) used to solve structure: *SHELXS97* (Sheldrick, 2008 ▶); program(s) used to refine structure: *SHELXS97* (Sheldrick, 2008 ▶); molecular graphics: *DIAMOND* (Brandenburg, 2006 ▶); software used to prepare material for publication: *SHELXTL* (Sheldrick, 2008 ▶).

## Supplementary Material

Crystal structure: contains datablock(s) I, global. DOI: 10.1107/S1600536813010234/zl2544sup1.cif

Structure factors: contains datablock(s) I. DOI: 10.1107/S1600536813010234/zl2544Isup2.hkl

Additional supplementary materials:  crystallographic information; 3D view; checkCIF report

## Figures and Tables

**Table 1:** Hydrogen-bond geometry (Å, °)

*D*—H⋯*A*	*D*—H	H⋯*A*	*D*⋯*A*	*D*—H⋯*A*
O2*W*—H2*WA*⋯O1	0.82 (2)	2.14 (2)	2.882 (5)	151 (5)
O1*W*—H1*WA*⋯O2*W*	0.85 (2)	1.87 (7)	2.623 (8)	147 (11)
N8—H8*A*⋯O1*W* ^i^	0.91	2.19	3.091 (7)	169

Symmetry code: (i) 
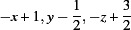
.

**Fig. 1.**
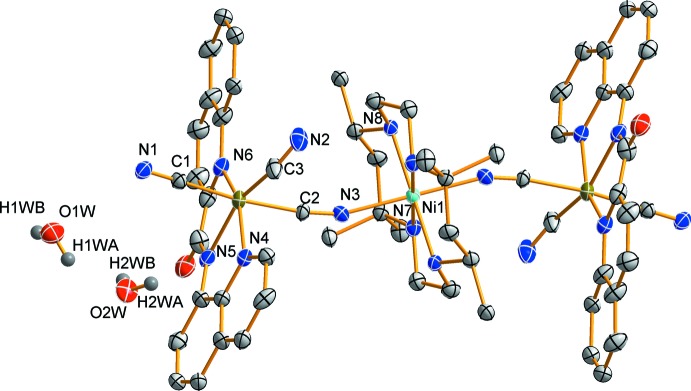
ORTEP diagram of the title complex with displacement ellipsoids drawn at the 30% probability level (The non-solvent H atoms have been omitted for clarity).

**Fig. 2.**
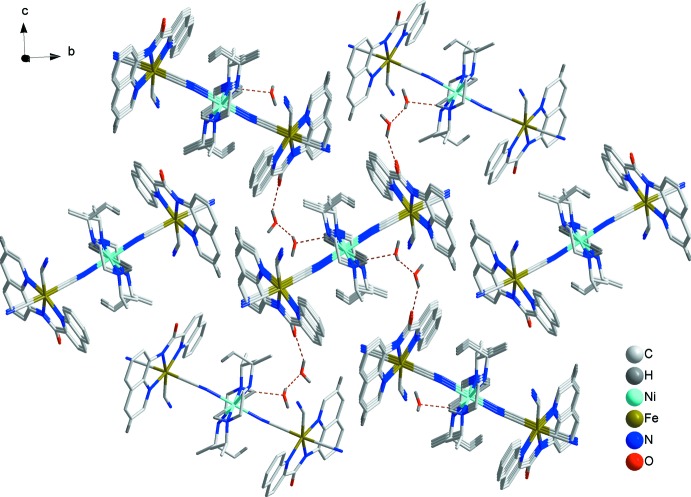
The packing and intermolecular interactions for the title complex (The dotted line represents the N—H···O and O—H···O hydrogen bonds).

## supplementary crystallographic information

### Comment

Modified hexacyanometalates, [Fe(qcq)(CN)_3_]^-^ (qcq^-^ = 8-(2-quinoline-2-carboxamido)quinoline anion) have been shown to be effective building blocks that can be used instead of hexacyanometalates for the design of low dimensional assemblies (Liu *et al.*, 2010). The capping ligand qcq^-^ (Li *et al.*, 2012) allows to limit oligomerization or polymerization effects by partially blocking the coordination sites around hexacyanometalates, and promotes the formation of low-dimensional structures. More importantly, it plays a crucial role in reducing the molecular symmetry, enhancing the anisotropy, and tuning the electronic, steric demand and solubility properties of derived complexes (Panja *et al.*, 2012). However, to the best of our knowledge, low dimensional compounds based on [Fe(qcq)(CN)_3_]^-^ as a ligand have been rarely explored and only a few related complexes have been reported so far. Therefore, the investigation of related low dimensional assemblies based on [Fe(qcq)(CN)_3_]^-^ is of significance. Considering that the macrocyclic cation of [Ni(teta)]^2+^ (teta = 5,5,7,12,12,14-hexamethyl-1,4,8,11-tetraazacyclotetradecane) can behave as a good electron acceptor, our synthesis strategy is to employ [Fe(qcq)(CN)_3_]^-^ and [Ni(teta)]^2+^ as precursors to construct low dimensional assemblies. Herein, the crystal structure of a new trinuclear complex, [{Ni(teta)}{Fe(qcq)(CN)_3_}_2_].2H_2_O is presented.

The molecular structure of the title complex is shown in Fig. 1. Within the neutral trinuclear clusters, two [Fe(qcq)(CN)_3_]^-^ anions coordinate to the central [Ni(teta)]^2+^ cation in a *trans*-mode, resulting in a nearly linear and centrosymmetric structure, where the Ni atom lies on an inversion centre. For the moieties of [Fe(qcq)(CN)_3_]^-^, the central Fe ion is coordinated by three C atoms from cyanide groups (Fe—C(cyanide) bond lengths: 1.951 (4)–1.965 (4) Å) and three N atoms from qcq^-^ (Fe—N(qcq) bond lengths: (1.970 (4)–2.146 (3) Å), affording a distorted octahedral coordination for the metal centre. The Fe—N (amide) bond length (1.970 (4) Å) is shorter than those for the Fe—N (aromatic rings) (2.045 (4)–2.146 (3) Å), which can be attributed to the strong σ-donor effect of the deprotonated amide. The bond angles of Fe1—C1—N1 and Fe1—C2—N3 remain almost linear (172.6 (3)–179.1 (4)°), while the Fe1—C3—N2 one deviates significantly from linearity (150.7 (5) °). The bond angle of Ni—N—C(cyanide) also deviates from linearity (161.1 (3)°), which is comparable to values observed in many other cyano-bridged bimetallic assemblies (Kim *et al.*, 2009). For the structural unit of [Ni(teta)]^2+^, the equatorial sites of the central Ni ion are occupied by four nitrogen atoms from the macrocyclic ligand of teta (Ni—N_macro_ bond lengths: 2.077 (3)–2.092 (3) Å), while the axial positions are occupied by N_cyanide_ from [Fe(qcq)(CN)_3_]^-^ (Ni—N_cyanide_ bond lengths: 2.116 (3) Å). The intramolecular Fe···Ni distance is 5.101 (3) Å. For the intermolecular interactions, the interstitial water molecules are positioned between the clusters and linked to the nitrogen atom of teta and the oxygen atom of adjacent clusters *via* hydrogen bonds, further extending the dimensionality of the structure to a supramolecular network, as shown in Fig. 2.

### Experimental

The complex was obtained as black block crystals by slow diffusion of a methanol solution (5 ml) of PPh_4_[Fe(qcq)(CN)_3_] (0.10 mmol) (Kim *et al.*, 2009) and a water/DMF (v:v = 7:8) solution (15 ml) of [Ni(teta)](ClO_4_)_2_ (0.10 mmol) (Curtis *et al.*, 1964) through a H-shaped tube at room temperature for about two weeks. The resulting crystals were collected, washed with H_2_O and CH_3_OH, respectively, and dried in air. Anal. found: C, 57.70; H, 5.23; N, 18.04; Fe, 8.92; Ni, 4.87%. Calcd for C_60_H_64.14_Fe_2_N_16_NiO_4.07_: C, 57.95; H, 5.19; N, 18.02; Fe, 8.98; Ni, 4.72%.

### Refinement

All non-H atoms were refined with anisotropic thermal parameters. The C– and N-bound H atoms were placed in idealized positions and included in the refinement in a riding mode (C—H = 0.95 Å, N—H = 0.88 Å) with *U*_iso_ for H assigned as 1.2 or 1.5 times *U*_eq_ of the attached atoms. The oxygen atoms (O1W, O2W) of interstitial water molecules are refined with partial occupancy factors of 0.528 (10) for the water molecule of O1W and 0.506 (9) for that of O2W, respectively. The water H-atoms were located from difference maps and were refined with a O—H and H···H distance restraints of 0.82 (2) Å) and 1.36 (2) Å and with *U*_iso_ for H assigned as 1.5 times *U*_eq_ of the attached atoms. The H atom H2WA was further restrained to be 2.10 (2) Å from O1 to rationalize the hydrogen bonds interactions.

### Crystal data

**Table:**

[Fe_2_Ni(C_19_H_12_N_3_O)_2_(CN)_6_(C_16_H_36_N_4_)]·2.07H_2_O	*F*(000) = 1297.4
*M**_r_* = 1244.89	*D*_x_ = 1.384 Mg m^−^^3^
Monoclinic, *P*2_1_/*c*	Mo *K*α radiation, λ = 0.71073 Å
Hall symbol: -P 2ybc	Cell parameters from 3735 reflections
*a* = 9.4145 (13) Å	θ = 2.1–23.4°
*b* = 15.7309 (17) Å	µ = 0.85 mm^−^^1^
*c* = 20.590 (2) Å	*T* = 291 K
β = 101.781 (3)°	Block, black
*V* = 2985.1 (6) Å^3^	0.28 × 0.24 × 0.22 mm
*Z* = 2	

### Data collection

**Table:**

Rigaku Saturn 724 CCD diffractometer	5722 independent reflections
Radiation source: fine-focus sealed tube	4078 reflections with *I* > 2σ(*I*)
Graphite monochromator	*R*_int_ = 0.022
φ and ω scans	θ_max_ = 26.0°, θ_min_ = 3.3°
Absorption correction: multi-scan (*ABSCOR*; Higashi, 1995)	*h* = −11→11
*T*_min_ = 0.796, *T*_max_ = 0.835	*k* = 0→19
12764 measured reflections	*l* = 0→25

### Refinement

**Table:**

Refinement on *F*^2^	Primary atom site location: structure-invariant direct methods
Least-squares matrix: full	Secondary atom site location: difference Fourier map
*R*[*F*^2^ > 2σ(*F*^2^)] = 0.057	Hydrogen site location: inferred from neighbouring sites
*wR*(*F*^2^) = 0.156	H atoms treated by a mixture of independent and constrained refinement
*S* = 0.97	*w* = 1/[σ^2^(*F*_o_^2^) + (0.095*P*)^2^] where *P* = (*F*_o_^2^ + 2*F*_c_^2^)/3
5722 reflections	(Δ/σ)_max_ < 0.001
402 parameters	Δρ_max_ = 0.42 e Å^−^^3^
7 restraints	Δρ_min_ = −0.42 e Å^−^^3^

### Special details

**Table:**

Geometry. All e.s.d.'s (except the e.s.d. in the dihedral angle between two l.s. planes) are estimated using the full covariance matrix. The cell e.s.d.'s are taken into account individually in the estimation of e.s.d.'s in distances, angles and torsion angles; correlations between e.s.d.'s in cell parameters are only used when they are defined by crystal symmetry. An approximate (isotropic) treatment of cell e.s.d.'s is used for estimating e.s.d.'s involving l.s. planes.
Refinement. Refinement of *F*^2^ against ALL reflections. The weighted *R*-factor *wR* and goodness of fit *S* are based on *F*^2^, conventional *R*-factors *R* are based on *F*, with *F* set to zero for negative *F*^2^. The threshold expression of *F*^2^ > σ(*F*^2^) is used only for calculating *R*-factors(gt) *etc*. and is not relevant to the choice of reflections for refinement. *R*-factors based on *F*^2^ are statistically about twice as large as those based on *F*, and *R*- factors based on ALL data will be even larger. the restraints that were used for the refinement of the water H atoms are: *DFIX* 2.1 H2WA O1 *DFIX* 1.36 H1WA H1WB H2WA H2WB *DFIX* 0.82 O1W H1WA O1W H1WB O2W H2WA O2W H2WB

### Fractional atomic coordinates and isotropic or equivalent isotropic displacement parameters (Å^2^)

**Table:**

	*x*	*y*	*z*	*U*_iso_*/*U*_eq_	Occ. (<1)
C1	0.6080 (3)	0.3867 (2)	0.85132 (16)	0.0385 (8)	
C2	0.4738 (3)	0.1784 (2)	0.92238 (15)	0.0381 (8)	
C3	0.6764 (4)	0.2943 (3)	0.9657 (2)	0.0603 (12)	
C4	0.4671 (4)	0.3845 (2)	1.00163 (18)	0.0439 (8)	
H4	0.5580	0.3678	1.0250	0.053*	
C5	0.3788 (4)	0.4394 (3)	1.03091 (17)	0.0475 (10)	
H5	0.4135	0.4595	1.0737	0.057*	
C6	0.2454 (4)	0.4634 (3)	0.99820 (18)	0.0535 (10)	
H6	0.1895	0.4999	1.0182	0.064*	
C7	0.1919 (4)	0.4330 (3)	0.93380 (19)	0.0507 (10)	
C8	0.0562 (4)	0.4568 (3)	0.89958 (18)	0.0479 (9)	
H8	−0.0011	0.4932	0.9188	0.057*	
C9	0.0051 (4)	0.4247 (3)	0.83428 (19)	0.0556 (11)	
H9	−0.0875	0.4387	0.8113	0.067*	
C10	0.0903 (4)	0.3742 (3)	0.80565 (18)	0.0480 (9)	
H10	0.0570	0.3549	0.7626	0.058*	
C11	0.2293 (4)	0.3504 (3)	0.84038 (18)	0.0492 (9)	
C12	0.2803 (3)	0.3806 (3)	0.90398 (17)	0.0431 (8)	
C13	0.3175 (4)	0.2439 (3)	0.76782 (19)	0.0531 (10)	
C14	0.4306 (4)	0.2005 (3)	0.76050 (17)	0.0470 (9)	
C15	0.4349 (4)	0.1491 (3)	0.70543 (19)	0.0535 (10)	
H15	0.3499	0.1343	0.6759	0.064*	
C16	0.5658 (4)	0.1213 (3)	0.6960 (2)	0.0601 (11)	
H16	0.5689	0.0894	0.6583	0.072*	
C17	0.6919 (4)	0.1378 (3)	0.73891 (19)	0.0549 (10)	
C18	0.8243 (4)	0.1087 (3)	0.7271 (2)	0.0565 (11)	
H18	0.8272	0.0769	0.6893	0.068*	
C19	0.9535 (4)	0.1276 (3)	0.7727 (2)	0.0536 (10)	
H19	1.0420	0.1080	0.7652	0.064*	
C20	0.9488 (4)	0.1744 (3)	0.8271 (2)	0.0539 (10)	
H20	1.0342	0.1857	0.8576	0.065*	
C21	0.8156 (4)	0.2063 (3)	0.8384 (2)	0.0534 (10)	
H21	0.8136	0.2406	0.8750	0.064*	
C22	0.6895 (4)	0.1863 (3)	0.79498 (18)	0.0462 (9)	
C23	0.3339 (3)	−0.0867 (2)	0.86776 (16)	0.0393 (8)	
C24	0.4859 (4)	−0.1054 (3)	0.85829 (17)	0.0447 (9)	
H24A	0.5204	−0.1545	0.8855	0.054*	
H24B	0.4782	−0.1227	0.8125	0.054*	
C25	0.6028 (4)	−0.0390 (2)	0.87275 (16)	0.0393 (8)	
H25	0.5618	0.0160	0.8561	0.047*	
C26	0.7755 (4)	0.0258 (3)	0.96768 (17)	0.0453 (9)	
H26A	0.7525	0.0793	0.9444	0.054*	
H26B	0.8632	0.0035	0.9562	0.054*	
C27	0.1984 (4)	−0.0414 (3)	0.95781 (16)	0.0449 (9)	
H27A	0.1243	−0.0845	0.9453	0.054*	
H27B	0.1649	0.0106	0.9342	0.054*	
C28	0.2337 (4)	−0.1648 (3)	0.84162 (17)	0.0476 (9)	
H28A	0.1373	−0.1542	0.8483	0.071*	
H28B	0.2716	−0.2150	0.8655	0.071*	
H28C	0.2312	−0.1728	0.7952	0.071*	
C29	0.2665 (4)	−0.0076 (3)	0.82503 (19)	0.0472 (9)	
H29A	0.2796	0.0425	0.8522	0.071*	
H29B	0.1648	−0.0171	0.8085	0.071*	
H29C	0.3142	−0.0003	0.7884	0.071*	
C30	0.7320 (4)	−0.0618 (3)	0.83749 (18)	0.0462 (9)	
H30A	0.7018	−0.1060	0.8054	0.069*	
H30B	0.8137	−0.0811	0.8700	0.069*	
H30C	0.7589	−0.0123	0.8156	0.069*	
Ni1	0.5000	0.0000	1.0000	0.0353 (2)	
Fe1	0.52931 (5)	0.28363 (4)	0.88333 (2)	0.04223 (18)	
N1	0.6534 (3)	0.4476 (2)	0.83299 (14)	0.0454 (7)	
N2	0.7764 (3)	0.2701 (3)	1.00090 (15)	0.0622 (10)	
N3	0.4567 (3)	0.1146 (2)	0.94597 (14)	0.0420 (7)	
N4	0.4158 (3)	0.3561 (2)	0.93779 (15)	0.0461 (8)	
N5	0.3195 (3)	0.2951 (2)	0.81931 (14)	0.0445 (7)	
N6	0.5568 (3)	0.2176 (2)	0.80568 (15)	0.0478 (8)	
N7	0.3381 (3)	−0.0703 (2)	0.93949 (14)	0.0412 (7)	
H7	0.3509	−0.1232	0.9576	0.049*	
N8	0.6599 (3)	−0.0325 (2)	0.94730 (12)	0.0363 (6)	
H8A	0.6936	−0.0848	0.9616	0.044*	
O1	0.2120 (3)	0.2298 (2)	0.72506 (14)	0.0600 (8)	
O1W	0.2133 (5)	0.2873 (4)	0.5217 (3)	0.066 (2)	0.528 (10)
H1WA	0.142 (7)	0.280 (7)	0.540 (4)	0.100*	0.528 (10)
H1WB	0.186 (9)	0.273 (8)	0.4823 (19)	0.100*	0.528 (10)
O2W	0.0492 (5)	0.2088 (4)	0.5914 (2)	0.059 (2)	0.506 (9)
H2WA	0.089 (9)	0.196 (3)	0.6296 (15)	0.088*	0.506 (9)
H2WB	0.051 (11)	0.177 (4)	0.562 (2)	0.088*	0.506 (9)

### Atomic displacement parameters (Å^2^)

**Table:**

	*U*^11^	*U*^22^	*U*^33^	*U*^12^	*U*^13^	*U*^23^
C1	0.0440 (16)	0.032 (2)	0.0422 (18)	0.0046 (15)	0.0141 (14)	0.0035 (16)
C2	0.0486 (17)	0.040 (2)	0.0293 (16)	0.0066 (15)	0.0166 (13)	0.0102 (15)
C3	0.0380 (17)	0.065 (3)	0.071 (3)	−0.0173 (17)	−0.0054 (17)	0.033 (2)
C4	0.0407 (15)	0.039 (2)	0.053 (2)	0.0031 (16)	0.0106 (14)	0.0069 (17)
C5	0.0520 (18)	0.053 (3)	0.0423 (18)	−0.0232 (17)	0.0210 (15)	−0.0186 (18)
C6	0.064 (2)	0.053 (3)	0.050 (2)	0.013 (2)	0.0258 (18)	0.015 (2)
C7	0.0521 (19)	0.041 (2)	0.063 (2)	0.0119 (17)	0.0223 (17)	0.0165 (19)
C8	0.0507 (18)	0.049 (3)	0.049 (2)	0.0148 (17)	0.0218 (16)	0.0165 (19)
C9	0.0426 (17)	0.070 (3)	0.057 (2)	0.0203 (18)	0.0152 (16)	0.028 (2)
C10	0.0452 (17)	0.051 (3)	0.0476 (19)	−0.0102 (17)	0.0088 (15)	−0.0018 (18)
C11	0.0509 (18)	0.047 (3)	0.053 (2)	0.0161 (17)	0.0185 (16)	0.0152 (19)
C12	0.0443 (17)	0.043 (2)	0.0457 (19)	0.0084 (16)	0.0187 (15)	0.0112 (17)
C13	0.066 (2)	0.047 (3)	0.043 (2)	−0.0011 (19)	0.0043 (18)	−0.0028 (19)
C14	0.0513 (18)	0.052 (3)	0.0377 (18)	−0.0157 (17)	0.0102 (14)	−0.0001 (17)
C15	0.0537 (19)	0.054 (3)	0.054 (2)	−0.0229 (18)	0.0128 (16)	−0.017 (2)
C16	0.054 (2)	0.060 (3)	0.069 (3)	−0.002 (2)	0.0193 (19)	−0.004 (2)
C17	0.058 (2)	0.057 (3)	0.056 (2)	−0.0156 (19)	0.0268 (18)	−0.015 (2)
C18	0.062 (2)	0.059 (3)	0.056 (2)	−0.009 (2)	0.0284 (19)	−0.018 (2)
C19	0.0438 (18)	0.055 (3)	0.065 (2)	0.0123 (17)	0.0201 (17)	0.018 (2)
C20	0.0451 (17)	0.055 (3)	0.065 (2)	0.0106 (17)	0.0180 (17)	0.023 (2)
C21	0.0485 (18)	0.046 (3)	0.072 (3)	0.0142 (17)	0.0268 (18)	0.021 (2)
C22	0.0518 (19)	0.043 (2)	0.050 (2)	−0.0107 (17)	0.0247 (16)	−0.0084 (18)
C23	0.0411 (15)	0.035 (2)	0.0445 (18)	0.0026 (14)	0.0162 (14)	0.0054 (16)
C24	0.0525 (18)	0.044 (2)	0.0407 (18)	0.0126 (17)	0.0179 (15)	−0.0069 (17)
C25	0.0500 (17)	0.036 (2)	0.0348 (16)	0.0095 (15)	0.0165 (14)	0.0013 (16)
C26	0.0458 (17)	0.044 (2)	0.050 (2)	0.0035 (16)	0.0183 (15)	−0.0004 (18)
C27	0.0433 (16)	0.050 (3)	0.0411 (18)	0.0061 (16)	0.0088 (14)	0.0074 (18)
C28	0.0572 (19)	0.040 (2)	0.0416 (19)	−0.0095 (17)	0.0016 (15)	−0.0048 (17)
C29	0.0477 (19)	0.045 (3)	0.046 (2)	−0.0012 (15)	0.0013 (16)	0.0163 (17)
C30	0.0503 (17)	0.043 (2)	0.049 (2)	0.0150 (16)	0.0192 (15)	0.0126 (18)
Ni1	0.0407 (3)	0.0359 (4)	0.0322 (3)	0.0059 (2)	0.0141 (3)	0.0083 (3)
Fe1	0.0481 (3)	0.0385 (4)	0.0415 (3)	−0.0006 (2)	0.0123 (2)	0.0128 (2)
N1	0.0450 (14)	0.053 (2)	0.0410 (15)	−0.0037 (14)	0.0156 (12)	0.0039 (15)
N2	0.0565 (18)	0.075 (3)	0.0459 (18)	−0.0148 (18)	−0.0120 (15)	0.0167 (18)
N3	0.0428 (14)	0.037 (2)	0.0476 (17)	0.0098 (13)	0.0120 (12)	0.0068 (15)
N4	0.0432 (13)	0.049 (2)	0.0480 (17)	−0.0025 (14)	0.0137 (13)	0.0156 (16)
N5	0.0503 (15)	0.043 (2)	0.0461 (16)	−0.0048 (14)	0.0225 (13)	0.0056 (14)
N6	0.0488 (15)	0.045 (2)	0.0516 (17)	−0.0068 (14)	0.0159 (13)	0.0114 (15)
N7	0.0451 (14)	0.0350 (18)	0.0457 (16)	0.0004 (12)	0.0143 (12)	0.0065 (14)
N8	0.0425 (13)	0.0369 (18)	0.0326 (13)	0.0078 (12)	0.0145 (11)	0.0066 (13)
O1	0.0569 (15)	0.062 (2)	0.0602 (16)	−0.0231 (14)	0.0095 (13)	0.0007 (15)
O1W	0.052 (3)	0.072 (5)	0.074 (4)	−0.013 (3)	0.009 (3)	−0.005 (3)
O2W	0.054 (3)	0.073 (5)	0.039 (3)	−0.021 (3)	−0.017 (2)	−0.009 (3)

### Geometric parameters (Å, º)

**Table:**

C1—N1	1.144 (5)	C22—N6	1.401 (5)
C1—Fe1	1.952 (4)	C23—N7	1.492 (4)
C2—N3	1.140 (5)	C23—C24	1.513 (4)
C2—Fe1	1.958 (4)	C23—C28	1.576 (5)
C3—N2	1.131 (5)	C23—C29	1.579 (5)
C3—Fe1	1.965 (4)	C24—C25	1.503 (5)
C4—N4	1.378 (5)	C24—H24A	0.9700
C4—C5	1.416 (5)	C24—H24B	0.9700
C4—H4	0.9300	C25—N8	1.523 (4)
C5—C6	1.353 (5)	C25—C30	1.580 (4)
C5—H5	0.9300	C25—H25	0.9800
C6—C7	1.403 (6)	C26—N8	1.420 (5)
C6—H6	0.9300	C26—C27^i^	1.523 (5)
C7—C8	1.378 (5)	C26—H26A	0.9700
C7—C12	1.399 (5)	C26—H26B	0.9700
C8—C9	1.424 (6)	C27—N7	1.511 (4)
C8—H8	0.9300	C27—C26^i^	1.523 (5)
C9—C10	1.347 (6)	C27—H27A	0.9700
C9—H9	0.9300	C27—H27B	0.9700
C10—C11	1.408 (5)	C28—H28A	0.9600
C10—H10	0.9300	C28—H28B	0.9600
C11—N5	1.349 (5)	C28—H28C	0.9600
C11—C12	1.384 (5)	C29—H29A	0.9600
C12—N4	1.378 (4)	C29—H29B	0.9600
C13—O1	1.206 (5)	C29—H29C	0.9600
C13—C14	1.300 (6)	C30—H30A	0.9600
C13—N5	1.328 (5)	C30—H30B	0.9600
C14—N6	1.378 (5)	C30—H30C	0.9600
C14—C15	1.400 (5)	Ni1—N7^i^	2.079 (3)
C15—C16	1.359 (5)	Ni1—N7	2.079 (3)
C15—H15	0.9300	Ni1—N8^i^	2.091 (2)
C16—C17	1.352 (6)	Ni1—N8	2.091 (2)
C16—H16	0.9300	Ni1—N3^i^	2.114 (3)
C17—C22	1.388 (5)	Ni1—N3	2.114 (3)
C17—C18	1.395 (5)	Fe1—N6	1.967 (3)
C18—C19	1.409 (5)	Fe1—N4	2.047 (3)
C18—H18	0.9300	Fe1—N5	2.147 (3)
C19—C20	1.349 (6)	N7—H7	0.9100
C19—H19	0.9300	N8—H8A	0.9100
C20—C21	1.414 (5)	O1W—H1WA	0.85 (2)
C20—H20	0.9300	O1W—H1WB	0.83 (2)
C21—C22	1.369 (5)	O2W—H2WA	0.82 (2)
C21—H21	0.9300	O2W—H2WB	0.79 (2)
			
N1—C1—Fe1	179.3 (3)	N8—C26—H26B	109.5
N3—C2—Fe1	172.6 (3)	C27^i^—C26—H26B	109.5
N2—C3—Fe1	150.8 (5)	H26A—C26—H26B	108.0
N4—C4—C5	118.8 (3)	N7—C27—C26^i^	109.3 (3)
N4—C4—H4	120.6	N7—C27—H27A	109.8
C5—C4—H4	120.6	C26^i^—C27—H27A	109.8
C6—C5—C4	121.6 (3)	N7—C27—H27B	109.8
C6—C5—H5	119.2	C26^i^—C27—H27B	109.8
C4—C5—H5	119.2	H27A—C27—H27B	108.3
C5—C6—C7	119.5 (4)	C23—C28—H28A	109.5
C5—C6—H6	120.2	C23—C28—H28B	109.5
C7—C6—H6	120.2	H28A—C28—H28B	109.5
C8—C7—C12	120.5 (4)	C23—C28—H28C	109.5
C8—C7—C6	120.4 (4)	H28A—C28—H28C	109.5
C12—C7—C6	119.0 (3)	H28B—C28—H28C	109.5
C7—C8—C9	119.1 (4)	C23—C29—H29A	109.5
C7—C8—H8	120.5	C23—C29—H29B	109.5
C9—C8—H8	120.5	H29A—C29—H29B	109.5
C10—C9—C8	120.3 (3)	C23—C29—H29C	109.5
C10—C9—H9	119.8	H29A—C29—H29C	109.5
C8—C9—H9	119.8	H29B—C29—H29C	109.5
C9—C10—C11	120.6 (4)	C25—C30—H30A	109.5
C9—C10—H10	119.7	C25—C30—H30B	109.5
C11—C10—H10	119.7	H30A—C30—H30B	109.5
N5—C11—C12	114.0 (3)	C25—C30—H30C	109.5
N5—C11—C10	126.1 (4)	H30A—C30—H30C	109.5
C12—C11—C10	119.8 (3)	H30B—C30—H30C	109.5
N4—C12—C11	119.3 (3)	N7^i^—Ni1—N7	180.0
N4—C12—C7	121.0 (3)	N7^i^—Ni1—N8^i^	94.38 (11)
C11—C12—C7	119.7 (3)	N7—Ni1—N8^i^	85.62 (11)
O1—C13—C14	113.0 (4)	N7^i^—Ni1—N8	85.62 (11)
O1—C13—N5	124.7 (4)	N7—Ni1—N8	94.38 (11)
C14—C13—N5	122.2 (4)	N8^i^—Ni1—N8	179.998 (1)
C13—C14—N6	115.6 (4)	N7^i^—Ni1—N3^i^	95.69 (12)
C13—C14—C15	123.8 (4)	N7—Ni1—N3^i^	84.31 (12)
N6—C14—C15	119.8 (3)	N8^i^—Ni1—N3^i^	90.98 (11)
C16—C15—C14	118.6 (4)	N8—Ni1—N3^i^	89.02 (11)
C16—C15—H15	120.7	N7^i^—Ni1—N3	84.31 (12)
C14—C15—H15	120.7	N7—Ni1—N3	95.69 (12)
C17—C16—C15	123.1 (4)	N8^i^—Ni1—N3	89.02 (11)
C17—C16—H16	118.4	N8—Ni1—N3	90.98 (11)
C15—C16—H16	118.4	N3^i^—Ni1—N3	179.999 (1)
C16—C17—C22	119.2 (4)	C1—Fe1—C2	173.01 (14)
C16—C17—C18	121.3 (4)	C1—Fe1—C3	88.36 (15)
C22—C17—C18	119.5 (4)	C2—Fe1—C3	85.30 (15)
C17—C18—C19	119.7 (4)	C1—Fe1—N6	92.41 (13)
C17—C18—H18	120.2	C2—Fe1—N6	88.74 (13)
C19—C18—H18	120.2	C3—Fe1—N6	124.04 (17)
C20—C19—C18	119.9 (3)	C1—Fe1—N4	89.92 (13)
C20—C19—H19	120.0	C2—Fe1—N4	91.90 (13)
C18—C19—H19	120.0	C3—Fe1—N4	80.47 (16)
C19—C20—C21	120.8 (4)	N6—Fe1—N4	155.42 (12)
C19—C20—H20	119.6	C1—Fe1—N5	95.05 (13)
C21—C20—H20	119.6	C2—Fe1—N5	91.93 (13)
C22—C21—C20	119.4 (4)	C3—Fe1—N5	157.02 (16)
C22—C21—H21	120.3	N6—Fe1—N5	78.60 (12)
C20—C21—H21	120.3	N4—Fe1—N5	76.82 (12)
C21—C22—C17	120.7 (3)	C2—N3—Ni1	161.0 (3)
C21—C22—N6	119.9 (3)	C12—N4—C4	119.9 (3)
C17—C22—N6	119.4 (3)	C12—N4—Fe1	114.3 (2)
N7—C23—C24	109.1 (3)	C4—N4—Fe1	125.6 (2)
N7—C23—C28	111.5 (3)	C13—N5—C11	137.8 (3)
C24—C23—C28	108.6 (3)	C13—N5—Fe1	107.5 (2)
N7—C23—C29	110.0 (3)	C11—N5—Fe1	114.7 (2)
C24—C23—C29	111.4 (3)	C14—N6—C22	119.9 (3)
C28—C23—C29	106.2 (3)	C14—N6—Fe1	114.4 (2)
C25—C24—C23	120.8 (3)	C22—N6—Fe1	125.6 (2)
C25—C24—H24A	107.1	C23—N7—C27	116.8 (2)
C23—C24—H24A	107.1	C23—N7—Ni1	123.6 (2)
C25—C24—H24B	107.1	C27—N7—Ni1	105.0 (2)
C23—C24—H24B	107.1	C23—N7—H7	102.8
H24A—C24—H24B	106.8	C27—N7—H7	102.8
C24—C25—N8	109.9 (3)	Ni1—N7—H7	102.8
C24—C25—C30	110.6 (3)	C26—N8—C25	115.7 (3)
N8—C25—C30	109.4 (3)	C26—N8—Ni1	106.2 (2)
C24—C25—H25	109.0	C25—N8—Ni1	113.33 (18)
N8—C25—H25	109.0	C26—N8—H8A	107.1
C30—C25—H25	109.0	C25—N8—H8A	107.1
N8—C26—C27^i^	110.9 (3)	Ni1—N8—H8A	107.1
N8—C26—H26A	109.5	H1WA—O1W—H1WB	106 (3)
C27^i^—C26—H26A	109.5	H2WA—O2W—H2WB	120 (4)
			
N4—C4—C5—C6	1.1 (6)	N5—Fe1—N4—C4	176.8 (3)
C4—C5—C6—C7	0.4 (6)	O1—C13—N5—C11	−7.4 (8)
C5—C6—C7—C8	−179.9 (4)	C14—C13—N5—C11	175.9 (4)
C5—C6—C7—C12	−2.2 (6)	O1—C13—N5—Fe1	173.8 (4)
C12—C7—C8—C9	2.3 (6)	C14—C13—N5—Fe1	−3.0 (5)
C6—C7—C8—C9	180.0 (4)	C12—C11—N5—C13	172.3 (4)
C7—C8—C9—C10	−2.2 (6)	C10—C11—N5—C13	−3.6 (7)
C8—C9—C10—C11	1.7 (6)	C12—C11—N5—Fe1	−8.9 (4)
C9—C10—C11—N5	174.3 (4)	C10—C11—N5—Fe1	175.2 (3)
C9—C10—C11—C12	−1.4 (6)	C1—Fe1—N5—C13	99.2 (3)
N5—C11—C12—N4	3.1 (5)	C2—Fe1—N5—C13	−80.6 (3)
C10—C11—C12—N4	179.2 (3)	C3—Fe1—N5—C13	−163.1 (4)
N5—C11—C12—C7	−174.7 (4)	N6—Fe1—N5—C13	7.8 (3)
C10—C11—C12—C7	1.5 (6)	N4—Fe1—N5—C13	−172.1 (3)
C8—C7—C12—N4	−179.7 (3)	C1—Fe1—N5—C11	−80.0 (3)
C6—C7—C12—N4	2.6 (6)	C2—Fe1—N5—C11	100.3 (3)
C8—C7—C12—C11	−2.0 (6)	C3—Fe1—N5—C11	17.8 (5)
C6—C7—C12—C11	−179.7 (4)	N6—Fe1—N5—C11	−171.4 (3)
O1—C13—C14—N6	176.3 (3)	N4—Fe1—N5—C11	8.8 (3)
N5—C13—C14—N6	−6.6 (6)	C13—C14—N6—C22	−168.2 (4)
O1—C13—C14—C15	6.0 (6)	C15—C14—N6—C22	2.5 (5)
N5—C13—C14—C15	−176.9 (4)	C13—C14—N6—Fe1	13.6 (5)
C13—C14—C15—C16	166.2 (4)	C15—C14—N6—Fe1	−175.7 (3)
N6—C14—C15—C16	−3.7 (6)	C21—C22—N6—C14	177.3 (4)
C14—C15—C16—C17	3.0 (7)	C17—C22—N6—C14	−0.4 (6)
C15—C16—C17—C22	−0.9 (7)	C21—C22—N6—Fe1	−4.8 (5)
C15—C16—C17—C18	−179.4 (4)	C17—C22—N6—Fe1	177.6 (3)
C16—C17—C18—C19	179.6 (4)	C1—Fe1—N6—C14	−106.1 (3)
C22—C17—C18—C19	1.1 (7)	C2—Fe1—N6—C14	80.8 (3)
C17—C18—C19—C20	−0.6 (7)	C3—Fe1—N6—C14	164.3 (3)
C18—C19—C20—C21	−1.4 (6)	N4—Fe1—N6—C14	−11.0 (5)
C19—C20—C21—C22	2.9 (6)	N5—Fe1—N6—C14	−11.4 (3)
C20—C21—C22—C17	−2.4 (6)	C1—Fe1—N6—C22	75.9 (3)
C20—C21—C22—N6	−180.0 (3)	C2—Fe1—N6—C22	−97.2 (3)
C16—C17—C22—C21	−178.1 (4)	C3—Fe1—N6—C22	−13.7 (4)
C18—C17—C22—C21	0.5 (6)	N4—Fe1—N6—C22	171.0 (3)
C16—C17—C22—N6	−0.5 (6)	N5—Fe1—N6—C22	170.6 (3)
C18—C17—C22—N6	178.1 (4)	C24—C23—N7—C27	173.9 (3)
N7—C23—C24—C25	−64.1 (4)	C28—C23—N7—C27	−66.2 (4)
C28—C23—C24—C25	174.2 (3)	C29—C23—N7—C27	51.4 (4)
C29—C23—C24—C25	57.5 (4)	C24—C23—N7—Ni1	40.7 (4)
C23—C24—C25—N8	78.0 (4)	C28—C23—N7—Ni1	160.7 (2)
C23—C24—C25—C30	−161.2 (3)	C29—C23—N7—Ni1	−81.7 (3)
N2—C3—Fe1—C1	−113.9 (7)	C26^i^—C27—N7—C23	−178.6 (3)
N2—C3—Fe1—C2	63.2 (7)	C26^i^—C27—N7—Ni1	−37.6 (3)
N2—C3—Fe1—N6	−22.1 (7)	N8^i^—Ni1—N7—C23	150.3 (3)
N2—C3—Fe1—N4	156.0 (7)	N8—Ni1—N7—C23	−29.7 (3)
N2—C3—Fe1—N5	147.1 (6)	N3^i^—Ni1—N7—C23	−118.2 (3)
N7^i^—Ni1—N3—C2	33.8 (8)	N3—Ni1—N7—C23	61.8 (3)
N7—Ni1—N3—C2	−146.2 (8)	N8^i^—Ni1—N7—C27	12.8 (2)
N8^i^—Ni1—N3—C2	128.3 (9)	N8—Ni1—N7—C27	−167.2 (2)
N8—Ni1—N3—C2	−51.7 (9)	N3^i^—Ni1—N7—C27	104.2 (2)
C11—C12—N4—C4	−178.9 (3)	N3—Ni1—N7—C27	−75.8 (2)
C7—C12—N4—C4	−1.1 (5)	C27^i^—C26—N8—C25	167.7 (3)
C11—C12—N4—Fe1	4.6 (4)	C27^i^—C26—N8—Ni1	41.1 (3)
C7—C12—N4—Fe1	−177.7 (3)	C24—C25—N8—C26	177.5 (3)
C5—C4—N4—C12	−0.7 (5)	C30—C25—N8—C26	55.9 (4)
C5—C4—N4—Fe1	175.4 (3)	C24—C25—N8—Ni1	−59.6 (3)
C1—Fe1—N4—C12	88.3 (3)	C30—C25—N8—Ni1	178.8 (2)
C2—Fe1—N4—C12	−98.4 (3)	N7^i^—Ni1—N8—C26	−15.4 (2)
C3—Fe1—N4—C12	176.7 (3)	N7—Ni1—N8—C26	164.6 (2)
N6—Fe1—N4—C12	−7.3 (5)	N3^i^—Ni1—N8—C26	−111.1 (2)
N5—Fe1—N4—C12	−6.9 (2)	N3—Ni1—N8—C26	68.9 (2)
C1—Fe1—N4—C4	−88.0 (3)	N7^i^—Ni1—N8—C25	−143.4 (2)
C2—Fe1—N4—C4	85.3 (3)	N7—Ni1—N8—C25	36.6 (2)
C3—Fe1—N4—C4	0.4 (3)	N3^i^—Ni1—N8—C25	120.8 (2)
N6—Fe1—N4—C4	176.4 (3)	N3—Ni1—N8—C25	−59.2 (2)

Symmetry code: (i) −*x*+1, −*y*, −*z*+2.

### Hydrogen-bond geometry (Å, º)

**Table:**

*D*—H···*A*	*D*—H	H···*A*	*D*···*A*	*D*—H···*A*
O2*W*—H2*WA*···O1	0.82 (2)	2.14 (2)	2.882 (5)	151 (5)
O1*W*—H1*WA*···O2*W*	0.85 (2)	1.87 (7)	2.623 (8)	147 (11)
N8—H8*A*···O1*W*^ii^	0.91	2.19	3.091 (7)	169

Symmetry code: (ii) −*x*+1, *y*−1/2, −*z*+3/2.
